# Clinical correlates and cognitive associations of the neutrophil-to-lymphocyte ratio in first-episode psychosis and at-risk mental states

**DOI:** 10.3389/fpsyt.2026.1838805

**Published:** 2026-06-02

**Authors:** Clàudia Aymerich, Borja Pedruzo, Garazi Acasuso, Olatz Ibarretxe, Daniel Alonso-Alconada, Javier Labad, Maria José Algora, Ángel Cabezas, Gonzalo Salazar de Pablo, Miguel Ángel González-Torres, Vanessa Sanchez-Gistau, Ana Catalán

**Affiliations:** 1 Psychiatry Department, Basurto University Hospital, Biobizkaia Health Research Institute, OSI Bilbao-Basurto, Bilbao, Spain; 2 Centro de Investigación Biomédica en Red en Salud Mental (CIBERSAM), Madrid, Spain; 3 Department of Child and Adolescent Psychiatry, Institute of Psychiatry, Psychology & Neuroscience, King’s College London, London, United Kingdom; 4 Neuroscience Department. School of Medicine and Nursing, University of the Basque Country (EHU), Leioa, Spain; 5 Department of Cell Biology and Histology, School of Medicine and Nursing, University of the Basque Country (EHU), Leioa, Spain; 6 Department of Mental Health, Consorci Sanitari del Maresme, Mataró, Spain; 7 Institut de Neurociències, Translational Neuroscience Research Unit I3PT-INc-UAB, Institut d’Investigació I Innovació Parc Taulí (I3PT), Sabadell, Spain; 8 Hospital Universitari Institut Pere Mata, Reus, Spain; 9 Institut d’Investigació Sanitària Pere Virgili (IISPV-CERCA), Reus, Spain; 10 Universitat Rovira i Virgili (URV), Reus, Spain; 11 Institute of Psychiatry and Mental Health, Hospital General Universitario Gregorio Marañón School of Medicine, Universidad Complutense, IiSGM, Madrid, Spain; 12 Department of Psychosis Studies, Institute of Psychiatry, Psychology & Neuroscience, London, United Kingdom

**Keywords:** clinical high-risk for psychosis, inflammation, lymphocyte, neutrophil, psychosis, schizophrenia

## Abstract

**Objective:**

Clinical high-risk for psychosis (CHR-P) and first-episode psychosis (FEP) individuals present immune alterations that precede treatment initiation. The neutrophil-to-lymphocyte ratio (NLR) has emerged as a simple, accessible marker of systemic inflammation. This study investigates differences across CHR-P, FEP, and healthy control (HC) populations and explores peripheral associations between NLR and demographic, clinical, and cognitive variables.

**Methods:**

Data were collected from 63 FEP, 56 CHR-P, and 27 HC individuals from two early intervention services in Spain. Socio-demographic, clinical, and neurocognitive data were collected from all participants, along with peripheral blood samples for NLR calculation. Correlations between NLR and clinical and neurocognitive variables were analyzed using multivariate models to control for potential confounders.

**Results:**

No significant differences in NLR were observed between the FEP, CHR-P, and HC groups (F = 1.04; p=0.36). In the FEP population, NLR was positively correlated with higher levels of positive symptoms (β=0.035; p=0.01) and longer duration of untreated psychosis (β=0.003; p=0.04) after adjusting for sex and age. In CHR-P individuals, NLR was negatively correlated with antidepressant use (β=-0.664; p=0.02). No significant associations were found between NLR and neurocognitive performance or antipsychotic treatment in any clinical group.

**Conclusions:**

Our findings do not support the utility of NLR as a diagnostic biomarker in early psychosis. However, the observed association between elevated NLR and positive psychotic symptoms in FEP suggests that NLR could serve as a state biomarker, reflecting inflammatory status related to symptom severity. Further research is needed to explore NLR dynamics in larger samples and its potential role in monitoring clinical progression in psychosis.

## 1 Introduction

Schizophrenia is a heterogeneous disorder characterized by positive, negative, affective, and cognitive symptoms (1). Individuals with this disorder experience a reduction in life expectancy of 15 to 20 years (2), which has traditionally been attributed to the side effects of antipsychotic treatment (3) and the impact of the disorder itself on lifestyle (4). However, strong evidence points to dysfunctions across multiple systems in individuals with a first episode psychosis (FEP), occurring even before the introduction of treatment (5). Among these alterations, immune parameters stand out, with increased levels of pro-inflammatory interleukins compared to healthy controls (6, 7), with effect sizes exceeding those reported for brain structural alterations (5). There is also preliminary evidence of immune alterations even in the prodromal stages of psychosis (8).

In recent decades, the neutrophil-to-lymphocyte ratio (NLR), calculated as a simple ratio between neutrophil and lymphocyte counts in peripheral blood, has gained prominence as a straightforward, accessible, and inexpensive marker of systemic inflammation (9). An increase in the NLR has been shown to correlate with negative outcomes and predict disease in patients across a variety of medical conditions, including cancer (10), sepsis (11), cardiovascular events (12), and all-cause mortality in the general population (13).

In the field of schizophrenia, previous studies have reported an elevated NLR in both FEP samples (14, 15) and in multi-episode psychosis (16). This increase in NLR has also been correlated in FEP populations with elevated levels of other pro-inflammatory markers, such as prostaglandin PGE2 (17). However, the evidence is mixed regarding whether NLR is a disease marker or a state marker that may correlate with clinical features, such as the presence of more severe positive symptoms or depressive symptoms and fluctuate based on the individual’s clinical condition (18–20).

Only a limited number of studies have investigated inflammatory cell-derived markers in populations at clinical high-risk for psychosis (CHR-P), despite preliminary evidence of a pro-inflammatory state in this group (8, 21, 22).

Furthermore, there is no strong evidence to date on the relationship between NLR and neurocognitive impairments in psychosis populations. Both FEP (23) and CHR-P populations (24) show group-level impairments in neurocognitive functioning. There is evidence linking elevated NLR with cognitive dysfunction, both within Alzheimer’s disease (25) and in the early stages of mild cognitive impairment (26). In the case of psychotic disorders, however, while immune activation (measured through an increase in circulating cytokines) has been linked to poorer cognitive function (27), this could not be replicated in a study that aimed to associate it with the NLR (18), leaving significant questions unresolved.

Considering all the above, the aim of this study is a) to describe the differences in the NLR index between FEP, CHR-P, and HC groups; b) to analyze the relationship between the NLR index and neurocognitive profile across the three population groups; and c) to explore associations between NLR and demographic and clinical variables within the clinical populations.

We hypothesize that the NLR will be increased in a graded manner across groups, with the lowest values in HC, intermediate values in CHR-P, and the highest values in FEP. Furthermore, we hypothesize that higher NLR values will be associated with greater severity of psychotic symptoms and poorer neurocognitive functioning across the psychosis spectrum, supporting NLR as a marker of inflammation-related clinical and cognitive burden rather than a purely categorical disease marker.

## 2 Methods

### 2.1 Subjects

Data were collected from consecutive admissions of FEP and CHR-P individuals at the Early Intervention Service of Basurto University Hospital in Bilbao and the Early Intervention Service of Pere Mata University Hospital in Reus, Spain, between April 2016 and April 2024. HC subjects were recruited from the general population in the same catchment area as the Bilbao sample, using public announcements.

Inclusion criteria for all three groups were as follows: age between 16 and 45 years, sufficient proficiency in Spanish, IQ ≥ 70, and providing written informed consent. Additional criteria for the FEP group included meeting the definition of first-episode psychosis (patients presenting with psychosis, as per DSM (28, 29) or ICD (30, 31) criteria, and under antipsychotic treatment for less than 3 years). For the CHR-P group, participants had to meet criteria for one of the three established clinical high-risk mental states (32), as determined by validated psychometric instruments (**Supplementary Methods 1**). The CHR-P criteria include attenuated psychotic symptoms (APS), which involve mild positive symptoms; brief limited intermittent psychotic symptoms (BLIPS), characterized by short-lasting psychotic episodes that resolve spontaneously within a week; and genetic risk and deterioration syndrome (GRD), which applies to individuals with a family history of psychosis or schizotypal personality disorder, coupled with a recent decline in functioning (33).

Exclusion criteria for all groups included: any severe neurological, immune, or other significant physical diseases (including coronavirus disease); recent infection, fever, or use of anti-inflammatory drugs and/or antibiotics in the month prior to the enrollment; pregnancy or active breastfeeding; and substance use within the last month (excluding alcohol, tobacco, and cannabinoids). For the HC group, having a first-degree relative with a severe mental health disorder, a personal history of a severe mental health disorder, or being under active treatment with psychotropic medication for any reason – including antipsychotics, antidepressants, mood stabilizers, or benzodiazepines - was an additional exclusion criterion.

### 2.2 Procedures

#### 2.2.1 Socio-demographic and clinical measures

Socio-demographic, clinical, and neuropsychological variables were collected at study entry from each subject, including age, sex, self-reported ethnicity, years of education, and current use of antipsychotics (AP), antidepressants, mood stabilizers, and benzodiazepines (each defined as a dichotomous variable). In the FEP and CHR-P populations, the mean daily chlorpromazine equivalent dose of AP was also recorded, along with the duration of untreated psychosis (DUP) and the time elapsed between the blood test and the onset of psychotic symptoms and clinical evaluations.

The Positive and Negative Syndrome Scale (PANSS) (34), a widely used 30-item clinician-rated tool for assessing symptom severity in schizophrenia, was used to evaluate symptoms in FEP subjects. The Structured Interview for Psychosis-Risk Syndromes (SIPS) (35) and the Comprehensive Assessment of At-Risk Mental States (CAARMS) (36) were used to assess prodromal symptoms and states in CHR-P subjects. The validated conversion algorithm (CONVERT) was applied to derive SIPS scores from CAARMS scores for 3 participants (37). The Calgary Depression Scale for Schizophrenia (CDSS) (38), the only scale specifically designed to assess depression in individuals with schizophrenia spectrum disorder and attenuated psychotic symptoms, was used to evaluate depressive symptoms in the study subjects. The CDSS was administered across all study groups to maintain methodological consistency and comparability, including in healthy controls, who did not present current or past severe psychiatric disorders (39). The Global Assessment of Functioning (GAF) (40) scale was used to measure the subjects’ overall level of social functioning, considering impairments in psychological, social and occupational/academic functioning. The scale ranges from 0 to 100, with higher scores indicating better social functioning. Finally, in clinical subgroups, the Clinical Global Impression (CGI) (41) scale was also used to assess overall symptom severity.

#### 2.2.2 Neuropsychological measures

The Spanish adaptation of the Hopkins Verbal Learning Test – Revised (HVLT-R) (42) was used to assess verbal learning and memory. Participants were presented with a list of 12 items from three semantic categories across three trials, with the total score recorded. Higher scores indicated better performance.

The Animal Fluency test (43) assessed processing speed by asking subjects to name as many animals as possible within 60 seconds. The direct score was recorded, with higher scores reflecting better performance.

The Trail Making Test (TMT) (44) was used to measure attention and processing speed (part A, TMT-A) and executive function (part B, TMT-B). The total time in seconds for TMT-A and TMT-B was recorded, with higher scores indicating worse performance.

The Spanish adaptation of the Stroop Test (45) assessed inhibitory control and interference control, which is the ability to inhibit a habitual response in favor of a less familiar one. Three direct scores were recorded: the word reading score (Stroop-W) and the color naming score (Stroop-C) measured processing speed, while the color-word score (Stroop-WC) reflected the ability to inhibit automatic responses. Higher scores indicated better performance.

The Spanish adaptation of the Hinting Task (46), a widely used assessment for measuring social cognition in patients with schizophrenia, was used to assess mentalizing abilities. Subjects were presented with 10 short vignettes where one of the characters drops a hint to another. The direct score was recorded, with higher scores indicating better performance.

Finally, a premorbid IQ was estimated for all subjects based on the scores obtained from the Vocabulary subtest of the Wechsler Adult Intelligence Scale (WAIS-IV) (47).

#### 2.2.3 Blood cell analysis

Within four weeks of the clinical and cognition assessments, 5 mL of peripheral venous blood were collected from each subject in a fasting state and placed in an EDTA anticoagulation tube. The test results include WBC count, platelet count, neutrophil count, lymphocyte count, monocyte count, and eosinophil count. The NLR was calculated for each subject by dividing the total neutrophil count by the total lymphocyte count.

### 2.3 Statistical analysis

The Kolmogorov-Smirnov test with Lilliefors correction was used to assess the normality of the study variables. Descriptive analyses were conducted using frequency tables, means, and standard deviations (SD). Socio-demographic variables, clinical measures, neuropsychological test results, and blood count data were compared across the three study groups. For continuous variables, comparisons were made using one-way analysis of variance (ANOVA) or the Kruskal-Wallis test, depending on the data distribution. Categorical variables were compared using the Chi-Square test or the Fisher’s exact test.

Bivariate analyses were performed using Pearson’s correlation. NLR was considered the dependent variable, and each demographic, clinical, and neuropsychological variable was treated as an independent variable. Subsequently, a multivariate analysis model was conducted, including age and sex [two factors shown to be related to systemic inflammation (48, 49)], and study group as covariates.

This analysis was repeated for specific subgroups: the FEP subsample (incorporating variables such as the DUP and PANSS domains and total scores) and time since psychosis onset), the CHR-P subsample (with SIPS domains and total scores as dependent variables), and the clinical subsample (including both the FEP and the CHR-P populations).

The same procedure was used to investigate the relationship between the NLR and the other variables. A two-sided significance level of *p* < 0.05 was applied to all analyses. All statistical analyses were conducted using R software, version 4.2.2 (50).

### 2.4 Ethical compliance

The study was designed and conducted in accordance with the principles of the Declaration of Helsinki and received ethical approval from the Basurto University Hospital Research Ethics Committee (N.06.23 CEIHU), the Basque Country Research Ethics Committee (PI+CES-BIOEF 2023-03) and the Pere Virgili Health Research Institute Research Ethics Committee (233/2023).

## 3 Results

### 3.1 Subject characteristics

Sixty-three individuals with FEP, fifty-six with CHR-P, and twenty-seven HC were included in the present study. Socio-demographic, clinical, neurocognitive characteristics, and biological markers are presented in **Table 1**.

**Table 1:** Demographic, clinical, neurocognitive characteristics, and biological markers of the study sample.

Variable	FEP (N = 63)	CHR-P (N = 56)	HC (N = 27)	Statistic	*p* value
Age	25.47 (7.82)	20.48 (4.68)	27.20 (3.91)	F = 15.21	< 0.01*
% Female Sex	19 (30.16%)	33 (58.9%)	16 (59.3%)	X^2^ = 11.52	<0.01*
% White Ethnicity	46 (73.02%)	47 (83.9%)	26 (96.3%)	Fisher’s exact test	0.023
% Antipsychotic	59 (93.65%)	20 (35.7%)	0 (0%)	X^2^ = 80.21	<0.01*
Daily Chlorpromazine Equivalent Dose	300.00 (200.10)	54.91 (104.98)	0.00	t = -8.39	<0.01*
% Antidepressant	11 (17.46%)	23 (41.1%)	0 (0.0%)	X^2^ = 11.70	<0.01*
% Benzodiazepines	24 (38.1%)	13 (23.2%)	0 (0.0%)	X^2^ = 12.38	<0.01*
% Mood stabilizer	17 (26.98%)	6 (10.7%)	0 (0.0%)	X^2^ = 12.36	<0.01*
Education Years	11.22 (2.63)	10.85 (2.66)	15.70 (2.63)	F = 34.54	< 0.01*
Approximate IQ	90.60 (20.18)	103.72 (17.92)	99.10 (16.40)	F = 6.27	<0.01*
DUP (days)	48.25 (110.05)	n.a.	n.a.	n.a.	n.a.
PANSS - Positive	14.25 (7.35)	n.a.	n.a.	n.a.	n.a.
PANSS - Negative	19.36 (9.68)	n.a.	n.a.	n.a.	n.a.
PANSS - General	37.80 (16.59)	n.a.	n.a.	n.a.	n.a.
PANSS - Total	71.41 (29.61)	n.a.	n.a.	n.a.	n.a.
SIPS – Positive	n.a.	7.55 (4.61)	n.a.	n.a.	n.a.
SIPS – Negative	n.a.	10.85 (6.61)	n.a.	n.a.	n.a.
SIPS – Disorganization	n.a.	6.04 (3.73)	n.a.	n.a.	n.a.
SIPS – General	n.a.	8.87 (4.07)	n.a.	n.a.	n.a.
SIPS - Total	n.a.	33.32 (13.20)	n.a.	n.a.	n.a.
APS (%)	n.a.	42 (75.0%)	n.a.	n.a.	n.a.
BLIPS (%)	n.a.	6 (10.7%)	n.a.	n.a.	n.a.
GRD (%)	n.a.	11 (19.6%)	n.a.	n.a.	n.a.
GAF	55.34 (12.51)	59.43 (10.21)	84.01 (5.25)	F = 66.08	<0.01*
CDSS	3.32 (3.92)	4.92 (3.95)	0.65 (1.07)	F = 11.27	<0.01*
CGI	4.05 (1.05)	3.21 (0.99)	n.a.	t = -4.43	<0.01*
TMT-B (seconds)	93.63 (43.14)	72.71 (30.47)	56.92 (16.40)	F = 10.39	<0.01*
Stroop – Word	91.16 (18.06)	106.44 (20.52)	118.42 (17.00)	F = 20.27	<0.01*
Stroop - Colour	62.06 (12.76)	69.48 (9.90)	82.64 (12.51)	F = 25.99	<0.01*
Stroop – Word/Colour	38.98 (10.54)	46.96 (9.56)	55.69 (8.84)	F = 24.48	<0.01*
Hinting test	16.00 (3.10)	17.25 (2.33)	19.52 (1.35)	F = 16.48	<0.01*
TMT-A (seconds)	36.86 (12.45)	28.51 (9.85)	27.64 (6.72)	F = 11.46	<0.01*
HVLT-R	24.30 (4.73)	26.50 (4.07)	28.70 (3.88)	F = 10.71	<0.01*
Fluency (animal category)	18.68 (5.70)	24.27 (6.71)	28.43 (7.09)	F = 24.53	<0.01*
Total WBC (cells/µL)	7031.30 (1861.35)	7083.62 (1918.72)	6935.24 (1441.08)	F = 0.13	0.88
Neutrophil count (cells/µL)	3834.85 (1542.62)	3910.72 (1532.78)	4121.17 (1191.34)	X^2^ = 0.73	0.70
Lymphocyte count (cells/µL)	2367.59 (611.19)	2334.40 (742.63)	2130.01 (643.27)	F = 0.91	0.37
Monocyte count (cells/µL)	576.80 (165.92)	566.19 (185.49)	545.92 (134.31)	F = 0.25	0.78
Eosinophil count (cells/µL)	231.61 (150.54)	228.02 (191.02)	193.33 (138.46)	X^2^ = 1.82	0.40
NLR	1.71 (0.77)	1.84 (1.03)	2.06 (0.72)	F = 1.04	0.36

Results are expressed in mean (standard deviation) or number (%) when appropriate. For daily chlorpromazine equivalent dose, results are reported from an independent samples t-test comparing the two clinical groups (FEP and CHR-P), as the HC were not receiving antipsychotic medication. *indicates statistical significance (p value < 0.05). n.a., Not Applicable; IQ, Intelligence Quotient; DUP, Duration of Untreated Psychosis; PANSS, Positive and Negative Syndrome Scale; APS, Attenuated Psychotic Symptoms; BLIPS, Brief Limited Intermittent Psychotic Symptoms; GRD, Genetic Risk and Deterioration; GAF, Global Assessment of Functioning; CDSS, Calgary Depression Scale for Schizophrenia; CGI, Clinical Global Impression; TMT, Trail Making Test; HVLT-R, Hopkins Verbal Learning Test – Revised, WBC, White Blood Count; NLR, Neutrophil-to-Lymphocyte Ratio. Parametric tests included Student’s *t*-test and one-way ANOVA. Non-parametric analyses, when applicable, included the Mann–Whitney U test or Kruskal–Wallis test for quantitative variables, and the chi-square test for categorical variables.

The study groups differed significantly in most socio-demographic, clinical, and neurocognitive variables. The FEP group was, on average, older (F = 15.21; p < 0.01) and had a lower percentage of females (X2 = 11.52; p < 0.01) compared to the CHR-P and HC groups. Mean time of antipsychotic exposure was 173.25 days (SD = 246.45). The FEP group also demonstrated significantly greater use of antipsychotics, antidepressants, mood stabilizers, and benzodiazepines. Around 35% of the CHR-P sample were receiving antipsychotic treatment. However, in most cases, as reflected by the mean chlorpromazine equivalent dose, the dosage was significantly below the minimum antipsychotic threshold (51). In terms of neurocognitive functioning, both FEP and CHR-P groups exhibited lower performance than HC across all domains, with the FEP group performing worse overall. This includes impairments in executive function (measured with the Stroop test; F = 24.48; p < 0.01), social cognition (measured with the Hinting Task; F = 16.48; p < 0.01), and processing speed (measured with the verbal fluency test; F = 24.53; p < 0.01), among others.

No statistically significant differences were observed across the three study groups in the absolute lymphocyte, neutrophil, monocyte and eosinophil counts (all p values >0.05), and mean values were within the respective reference ranges (see **Supplementary Results 1**).

Likewise, NLR did not significantly differ between the three study groups (F = 1.04; p = 0.36), with no significant pairwise differences between HC and FEP (t = 1.64; p = 0.11) or between HC and CHR-P groups (t = 0.78; p = 0.44).

### 3.2 Associations of NLR with socio-demographic, clinical, and neurocognitive variables

Correlation analyses were conducted for both the full sample (FEP, CHR-P, and HC groups) and the clinical subsample (FEP and CHR-P groups). In both cases, elevated NLR was positively correlated with years of education and better performance on the TMT-A, TMT-B, and verbal fluency tests. However, none of these correlations remained statistically significant in the multivariate analysis after adjusting for age, sex, and study group. Detailed results for the full sample and the clinical subsample can be found in the **Supplementary Materials** (**Supplementary Results 2**, **3**, respectively).

Focusing exclusively on the FEP group, correlation analyses revealed a positive association between elevated NLR, years of education, longer DUP, and higher scores on the positive symptoms subscale of the PANSS. In the multivariable analysis, after adjusting for age and sex, all three variables remained statistically significant: education (β = 0.080; p = 0.04), DUP (β = 0.003; p = 0.04) and positive symptoms (β = 0.035; p = 0.01) (**Table 2**). No statistically significant correlations were found between NLR and other variables, including treatment, functioning, depressive symptoms, and neurocognitive performance.

**Table 2:** Results of the correlations between the neutrophil-to-lymphocyte ratio and other variables among the first-episode psychosis sample.

Variable	Univariate β	p value	Multivariate model
Age	0.177	0.20	n.a.
Male Sex	-0.023	0.91	n.a.
White Ethnicity	0.400	0.58	n.a.
Antipsychotic (Y/N)	-0.509	0.41	n.a.
Daily Chlorpromazine Equivalent Dose	0.145	0.30	n.a.
Antidepressant (Y/N)	0.136	0.54	n.a.
Benzodiazepines (Y/N)	-0.069	0.76	n.a.
Mood stabilizer (Y/N)	-0.139	0.62	n.a.
Education Years	0.319	0.02*	β = 0.080; p = 0.04*
Approximate IQ	-0.275	0.06	n.a.
DUP (days)	0.292	0.03*	β = 0.003; p = 0.04*
PANSS - Positive	0.305	0.03*	β = 0.035; p = 0.01*
PANSS - Negative	-0.045	0.75	n.a.
PANSS - General	0.171	0.22	n.a.
PANSS - Total	0.160	0.26	n.a.
GAF	-0.118	0.40	n.a.
CDSS	0.090	0.52	n.a.
CGI	0.153	0.27	n.a.
TMT-B (seconds)	-0.156	0.32	n.a.
Stroop – Word	0.067	0.63	n.a.
Stroop - Colour	-0.011	0.94	n.a.
Stroop – Word/Colour	0.119	0.39	n.a.
Hinting test	0.218	0.14	n.a.
TMT-A (seconds)	-0.239	0.08	n.a.
HVLT-R	0.033	0.81	n.a.
Fluency (animal category)	0.146	0.30	n.a.

*indicates statistical significance (p value < 0.05). n.a., Not Applicable; IQ, Intelligence Quotient; DUP, Duration of Untreated Psychosis; PANSS, Positive and Negative Syndrome Scale; GAF, Global Assessment of Functioning; CDSS, Calgary Depression Scale for Schizophrenia; CGI, Clinical Global Impression; TMT, Trail Making Test; HVLT-R, Hopkins Verbal Learning Test – Revised.

In contrast, in the CHR-P group, a negative correlation was observed between elevated NLR and antidepressant use, as well as poorer performance on the verbal fluency test scores. Although both variables showed a trend towards statistical significance, in the multivariable model, adjusted for age and sex, only antidepressant use remained statistically significant (β = -0.664; p = 0.02) (**Table 3**). However, no statistically significant correlations were found between NLR and depressive symptoms (measured with the CDSS), functioning, and neurocognitive performance.

**Table 3:** Results of the correlations between the neutrophil-to-lymphocyte ratio and other variables among the clinical high-risk for psychosis sample.

Variable	Univariate β	p value	Multivariate model
Age	0.181	0.22	n.a.
Male Sex	0.441	0.12	n.a.
White Ethnicity	0.337	0.74	n.a.
Antipsychotic (Y/N)	0.337	0.23	n.a.
Daily Chlorpromazine Equivalent Dose	0.034	0.82	n.a.
Antidepressant (Y/N)	0.564	0.04*	β = -0.664; p = 0.02*
Benzodiazepines (Y/N)	-0.239	0.45	n.a.
Mood stabilizer (Y/N)	-0.808	0.16	n.a.
Education Years	0.133	0.37	n.a.
Approximate IQ	0.045	0.78	n.a.
SIPS – Positive	0.381	0.21	n.a.
SIPS – Negative	0.032	0.83	n.a.
SIPS – Disorganization	0.128	0.42	n.a.
SIPS – General	-0.016	0.88	n.a.
SIPS - Total	0.217	0.29	n.a.
GAF	0.020	0.89	n.a.
CDSS	0.123	0.41	n.a.
TMT-B (seconds)	-0.287	0.06	n.a.
Stroop – Word	0.265	0.08	n.a.
Stroop - Colour	0.134	0.39	n.a.
Stroop – Word/Colour	0.175	0.26	n.a.
Hinting test	0.018	0.90	n.a.
TMT-A (seconds)	-0.215	0.15	n.a.
HVLT-R	0.141	0.35	n.a.
Fluency (animal category)	0.308	0.04*	β = 0.041; p = 0.06

*indicates statistical significance (p value < 0.05). n.a., Not Applicable; IQ, Intelligence Quotient; GAF, Global Assessment of Functioning; CDSS, Calgary Depression Scale for Schizophrenia; TMT, Trail Making Test; HVLT-R, Hopkins Verbal Learning Test – Revised.

## 4 Discussion

To the authors’ knowledge, this is one of the first comprehensive studies examining NLR and its correlates in the early stages of psychosis, including both FEP and CHR-P states. It also explores the association of this marker with socio-demographic, clinical, and neurocognitive factors across the different study groups.

Several interesting findings emerged from our study. First, contrary to our initial hypothesis, no significant differences in NLR were found between the FEP group and the HC group. Consistent with this, no between-group differences were observed in the absolute counts of any leukocyte subpopulation. Previous studies, including meta-analytical evidence (15, 16), have reported elevated NLR at a group level in FEP populations compared to HC (14), although some research has not found evidence of this correlation (52). A recent meta-analysis further reported increased neutrophil and monocyte counts in schizophrenia – particularly among patients not treated with antipsychotics – while other white blood cell subpopulations were not consistently altered (53). These discrepancies may be attributed to the limited statistical power in our analyses, although other studies have achieved statistical significance with sample sizes as small as 25 FEP subjects (54). Another possibility is the existence of different subgroups within the FEP population (e.g., those with higher levels of depressive symptoms or psychotic symptoms), which could account for these divergent results. Thus, an elevated NLR may serve as a state marker that fluctuates with the patient’s clinical condition, as suggested by previous studies (55), although longitudinal studies are required to confirm this hypothesis.

Our second finding supports this notion. In the FEP population, we observed a positive correlation between elevated NLR and higher scores on the PANSS positive symptom scale, which remained significant in multivariate models after adjusting for other statistically significant variables and socio-demographic factors. This is consistent with previous literature linking elevated NLR with higher levels of positive symptoms (18, 56). In line with this, a recent study found that patients with a psychotic disorder who fail to achieve symptomatic remission exhibit significantly higher NLR levels compared to those who remit, further supporting the link between inflammatory markers and illness severity (14). The relationship between these factors is complex and likely involves multiple psychopathological mechanisms. An increase in NLR reflects either a relative rise in neutrophil count, a reduction in lymphocyte count, or both. Both cell types play a crucial role in the immune dysregulation observed in schizophrenia. For instance, increased neutrophil levels in post-mortem samples have been linked to brain damage in neurodegenerative diseases (57). Interestingly, the other factor that was statistically significantly associated with an increase in NLR was DUP. A longer DUP has been consistently linked to negative long-term outcomes, including poorer general functioning, quality of life, and employment rates, among others (58). Our findings point to a potential involvement of inflammatory mechanisms, which could lead to increased neurotoxicity (59).

On the other hand, previous studies have overlooked the potential confounding effects of other psychopathological dimensions, particularly depressive states, on the neutrophil-to-lymphocyte ratio (NLR). However, in our study, we found no association between this parameter and depressive symptoms, as measured by the CDSS. Furthermore, the relationship between positive symptoms and a relative increase in NLR is independent of other demographic factors that could influence the inflammatory state, such as age (48) or sex (49). This suggests an independent association between elevated NLR and psychotic symptoms in FEP populations. Third, in the CHR-P population, no significant differences in NLR were detected compared to either the FEP or HC groups. Inflammatory alterations in individuals at clinical high risk for psychosis are a current topic of interest, with mixed results in the literature (60–62). Recent studies (22) using complementary methodologies, including DNA methylation-derived estimations of white blood cell proportions, have also suggested an association between immune alterations and clinical outcomes in CHR-P populations. While some studies have identified elevated pro-inflammatory interleukins (particularly IL-6 and IL-4) in this population (8), other studies have found no evidence of such alterations (62). It is plausible that immune changes in this subgroup are more subtle and variable than in established psychotic disorders, which may hinder clear alterations in systemic inflammatory markers such as NLR. Moreover, as with FEP, there may be distinct subgroups within the CHR-P population, whose group-level heterogeneity at all levels (63, 64) could mask these associations. Again, it is possible that NLR could serve as a state marker specific to certain subgroups within this population. Future research should focus on larger and longitudinal samples to validate these findings and explore longitudinal variables, such as the transition to a first episode of psychosis in CHR-P populations.

Fourth, no evidence was found for an association between elevated NLR and neurocognitive impairments in any of the studied domains. Notably, despite the absence of an association with NLR, clear group differences in neurocognitive functioning were observed: both clinical groups showed poorer performance compared to HC, and individuals with FEP consistently performed worse than those at clinical high risk. These differences were evident across all examined neurocognitive domains, consistently with what is described in the most recent meta-analysis on the topic (24). However, other studies have detected more heterogeneous patterns, detecting differences between CHR-P and HC groups in some cognitive domains but not in others, such as auditory or verbal learning (65). This variability may point to different vulnerability across specific cognitive processes.

The lack of an association between NLR and cognition is consistent with the previous study by Leung, that did not find any significant association between NLR and verbal memory or executive functions (18). This finding may appear paradoxical, as systemic inflammation – associated with elevated pro-inflammatory cytokines such as tumor necrosis factor β (TNF-β) and interferon γ (IFN-γ) – has been consistently associated with cognitive decline both in populations with established cognitive impairment (66) and in the general, non-clinical population (27), in both humans and animal models (67).

One possible explanation is that NLR represents a relatively coarse, peripheral marker of systemic inflammation and may not adequately capture central neuroinflammatory processes more directly implicated in cognitive functioning. Peripheral inflammatory alterations do not necessarily directly reflect neuroinflammatory mechanisms occurring within the central nervous system, which are likely more proximal to the pathophysiology of psychosis. Recent evidence also highlights the complex and only partially overlapping relationship between peripheral and central immune markers in psychotic disorders (68). In addition, inflammatory activity may influence cognition through indirect pathways, for example via its effects on symptom severity, affective disturbance, sleep, or overall illness burden, rather than through a direct linear relationship with neurocognitive performance. The pathophysiology of cognitive impairment in schizophrenia is clearly multifactorial, involving genetic and environmental factors that affect multiple neurotransmitter systems and mechanisms unrelated to inflammation (69). Some authors have even described that certain pro-inflammatory cytokines could play a protective role in neurocognition in FEP individuals (70). In this context, NLR may lack sufficient sensitivity to serve as a direct predictor of neurocognitive impairment during or after a FEP, despite the presence of marked global cognitive deficits in the clinical groups.

Lastly, the role of medication should also be considered in interpreting our results. AP use – a variable with a potential impact on the inflammatory status of individuals with psychotic disorders, though with conflicting results in recent literature (71) – was accounted for in multiple ways in this study, both through a dichotomous variable (particularly relevant in the CHR-P group, where only a minority of patients were on such treatment) and through analysis of daily chlorpromazine equivalents (with greater relevance to the FEP population). Neither variable was significantly associated with NLR. Due to the limited sample size and the heterogeneity of antipsychotic treatments received by participants, it was not possible to study the association of specific antipsychotics with NLR. Some evidence suggests specific anti-inflammatory profiles of certain antipsychotics, such as risperidone (72). Furthermore, it cannot be ruled out that previous studies linking a reduction in NLR after AP treatment in populations with schizophrenia may be mediated by a reduction in positive symptoms.

Additionally, the use of antidepressants in the CHR-P group showed a negative correlation with NLR. There is extensive evidence that individuals with depressive disorders exhibit elevated inflammatory markers (73, 74), including NLR (75, 76). Therefore, it is reasonable to hypothesize that the use of antidepressants – and the effective treatment of depressive symptoms – could have an anti-inflammatory effect. In this case, NLR could again serve as a state biomarker related to the patient’s clinical condition.

The findings of this study should be considered in light of its limitations. First, the sample size was relatively small and the study groups were unbalanced, which may have limited the statistical power to detect differences in some variables, including significant differences in NLR between study groups. In addition, the FEP group showed a relatively low proportion of female participants. Given evidence suggesting sex-related differences in inflammatory markers (77), this imbalance may have influenced the results. However, the sample size did not allow for meaningful sex-stratified analyses, and this should be addressed in future studies with larger samples. The FEP and CHR-P populations were also heterogeneous in terms of psychotropic exposure, including the duration and type of antipsychotic treatment. While antipsychotic dose was not significantly associated with NLR, the sample size limits the investigation of the effect of specific antipsychotics. In addition, blood sampling and clinical/neurocognitive assessments were conducted within a four-week time window rather than simultaneously. Considering that inflammatory markers may fluctuate over time and may be sensitive to changes in clinical state, this may have contributed to variability and reduced the ability to detect significant associations. Second, although no significant difference in NLR was found between CHR-P and HC groups, the impact of transition to psychosis on this marker could not be examined due to the lack of longitudinal data. Finally, while every effort was made to minimize confounding by excluding participants with medical conditions, active inflammatory states, recent infections, and recent use of anti-inflammatory medication or antibiotics, other potential confounders – such as obesity or tobacco use – could not be fully controlled.

In conclusion, the utility of the NLR index as a diagnostic biomarker for individuals in the early stages of psychosis, including CHR-P states and FEP is still unclear. However, a significant association was found between elevated NLR and higher positive symptom levels in FEP, suggesting a potential role as a state biomarker for monitoring treatment and clinical progression in this population. These findings should be replicated in larger samples with longitudinal data to explore the effects of variables such as the transition to a FEP in CHR-P groups.

## Data Availability

The raw data supporting the conclusions of this article will be made available by the authors, without undue reservation.
