# Supplementary material for: Clinical correlates and cognitive associations of the neutrophil-to-lymphocyte ratio in first-episode psychosis and at-risk mental states
Source: Front Psychiatry. 2026 Jun 2;17:1838805. doi: 10.3389/fpsyt.2026.1838805 (PMC13270933; doi:10.3389/fpsyt.2026.1838805)
Supplement: Supplementary file 1 [file SupplementaryFile1.docx]

**SUPPLEMENTARY MATERIAL**

**eMethods 1**: Types of CHR-P assessments included………………………………………….…….page 2

**eResults 2**: Results of the univariate correlations between the neutrophile-to-lymphocyte ratio and other variables among the full sample………………………………….…………………….…….page 3

**eResults 3**: Results of the univariate correlations between the neutrophile-to-lymphocyte ratio and other variables among the clinical sample………………………………….……..……….…….page 5

**eMethods 1**: Types of CHR-P assessments included (modified from Fusar-Poli et al., 2020)

- The following CHR-P instruments were considered to define the CHR-P state: Comprehensive Assessment of At-Risk Mental States (CAARMS) and Structured Interview for Psychosis-risk Syndromes (SIPS).
- Transition to psychosis was operationalised as defined by each CHR-P instruments or according to ICD/DSM criteria.

**eResults 1**: Reference values for white blood cell absolute counts.

| **Parameter** | **Reference range (cells / µL)** |
| --- | --- |
| Neutrophils | 1,500 – 7,500 |
| Lymphocytes | 1,000 – 4,000 |
| Monocytes | 200 – 800 |
| Eosinophiles | 0 – 500 |
| Total white blood cell count | 4,000 – 11,000 |

**eResults 2**: Results of the univariate correlations between the neutrophile-to-lymphocyte ratio and other variables among the full sample (including first-episode psychotic individuals, clinical high-risk for psychosis individuals, and healthy controls).

| **Variable** | **Univariate β** | **p Value** | **Multivariate model 1** | **Multivariate model 2** |
| --- | --- | --- | --- | --- |
| **Age** | 0.118 | 0.19 | N.a. | N.a. |
| **Male Sex** | 0.310 | 0.05* | β = -0.285; p = 0.10 | β = -0.286; p = 0.11 |
| **Race** | 0.727 | 0.42 | N.a. | N.a. |
| **Study Group** | 0.791 | 0.36 | N.a. | N.a. |
| **Education** | 0.195 | 0.02* | β = 0.030; p = 0.34 | β = 0.025; p = 0.54 |
| **GAF** | 0.055 | 0.55 | N.a. | N.a. |
| **CDSS** | 0.066 | 0.47 | N.a. | N.a. |
| **TMT-B** | -0.185 | 0.05* | β = -0.001; p = 0.77 | β = -0.001; p = 0.73 |
| **Stroop – Word** | 0.165 | 0.07 | N.a. | N.a. |
| **Stroop – Color** | 0.051 | 0.58 | N.a. | N.a. |
| **Stroop – Word / Color** | 0.141 | 0.12 | N.a. | N.a. |
| **Aprox IQ** | -0.001 | 0.99 | N.a. | N.a. |
| **Hinting Task** | 1.162 | 0.08 | N.a. | N.a. |
| **TMT-A** | -0.227 | 0.01* | β = -0.008; p = 0.36 | β = -0.009; p = 0.36 |
| **HVLT-R** | 0.146 | 0.11 | N.a. | N.a. |
| **Fluency Animal** | 0.089 | 0.02* | β = 0.012; p = 0.35 | β = 0.015; p = 0.29 |

* Indicates statistical significance (p value < 0.05). GAF Global Assessment of Functioning; CDSS Calgary Depression Scale for Schizophrenia; CTQ Childhood Trauma Questionnaire; TMT Trail Making Test; IQ Intelligence Quotient; HVLT-R Hopkins Verbal Learning Test – Revised.

**eResults 3**: Results of the univariate correlations between the neutrophile-to-lymphocyte ratio and other variables among the clinical sample (including first-episode psychotic individuals and clinical high-risk for psychosis individuals).

| **Variable** | **Univariate β** | **p Value** | **Multivariate model 1** | **Multivariate model 2** |
| --- | --- | --- | --- | --- |
| **Age** | 0.109 | 0.28 | N.a. | N.a. |
| **Male Sex** | 0.221 | 0.23 | N.a. | N.a. |
| **Race** | 0.565 | 0.56 | N.a. | N.a. |
| **Study Group** | 0.468 | 0.45 | N.a. | N.a. |
| **Education** | 0.215 | 0.03* | β = 0.053; p = 0.20 | β = 0.043; p = 0.36 |
| **GAF** | -0.037 | 0.71 | N.a. | N.a. |
| **CDSS** | 0.120 | 0.23 | N.a. | N.a. |
| **TMT-B** | -0.216 | 0.04* | β = -0.003; p = 0.33 | β = -0.002; p = 0.46 |
| **Stroop – Word** | 0.192 | 0.06 | N.a. | N.a. |
| **Stroop – Color** | 0.073 | 0.48 | N.a. | N.a. |
| **Stroop – Word / Color** | 0.163 | 0.11 | N.a. | N.a. |
| **Aprox IQ** | -0.063 | 0.56 | N.a. | N.a. |
| **Hinting Task** | 0.134 | 0.20 | N.a. | N.a. |
| **TMT-A** | -0.232 | 0.02* | β = -0.003; p = 0.73 | β = -0.005; p = 0.62 |
| **HVLT-R** | 0.100 | 0.32 | N.a. | N.a. |
| **Fluency Animal** | 0.251 | 0.01* | β = 0.020; p = 0.18 | β = 0.024; p = 0.15 |
| **Antipsychotic Dichotomic** | 0.181 | 0.39 | N.a. | N.a. |
| **Antidepressant Dichotomic** | 0.316 | 0.06 | N.a. | N.a. |
| **Eutimizer Dichotomic** | -0.228 | 0.37 | N.a. | N.a. |
| **Benzodiazepine Dichotomic** | -0.104 | 0.57 | N.a. | N.a. |
| **Chlorpromazine Equivalent Daily Dose** | 0.019 | 0.85 | N.a. | N.a. |

* Indicates statistical significance (p value < 0.05). * Indicates statistical significance (p value < 0.05). GAF Global Assessment of Functioning; CDSS Calgary Depression Scale for Schizophrenia; CTQ Childhood Trauma Questionnaire; TMT Trail Making Test; IQ Intelligence Quotient; HVLT-R Hopkins Verbal Learning Test – Revised
